# Beyond traditional teaching: a systematic review of innovative pedagogical practices in higher education

**DOI:** 10.12688/f1000research.143392.1

**Published:** 2024-01-08

**Authors:** Josefina Amanda Suyo-Vega, Víctor Hugo Fernández-Bedoya, Monica Elisa Meneses-La-Riva

**Affiliations:** 1 Grupo de Investigación “Educación Virtual, Universidad Cesar Vallejo, Lima Norte, Lima, 15314, Peru

**Keywords:** Pedagogical practices, Innovative pedagogical practices, Teaching strategies, Academic success, Higher education

## Abstract

**Background:**

Pedagogical best practices play a pivotal role in ensuring the academic success of students within the higher education landscape. This study aims to systematically synthesize innovative pedagogical best practices within the university context.

**Methods:**

We conducted a thorough systematic review using the rigorous PRISMA (Preferred Reporting Items for Systematic Reviews and Meta-Analyses) methodology. Our review involved comprehensive searches of scientific databases, including Eric, Scopus, and Proquest, covering both Spanish and English publications. We strategically employed Boolean operators like AND and OR to create a robust search equation. Our primary research question guiding this investigation was: “What innovative pedagogical practices have been developed in university settings to improve teaching and learning effectiveness?” This central question led us to delve deeply into the strategies utilized, pedagogical approaches adopted, and the noticeable impact achieved after their implementation. Additionally, we carefully established stringent inclusion and exclusion criteria in accordance with PRISMA guidelines to ensure methodological rigor.

**Results:**

A total of 25 scholarly articles that met the pre-established criteria were meticulously identified and included in this systematic review. The results were thoughtfully categorized into three distinct teaching strategies: the first emphasizing student-centered approaches, the second showcasing the integration of educational technology, and the third highlighting evaluation and feedback methodologies.

**Conclusions:**

This systematic exploration of pedagogical best practices underscores their paramount importance in driving continuous improvement in teacher training and fostering innovation within the educational arena. Such practices not only create an engaging and effective learning environment but also set the stage for ongoing advancements in the teaching and learning processes.

## Introduction

In the university system, the ability to provide quality education is the highest expression that is achieved through the faculty. In this regard, the good practices developed by university professors become essential elements to ensure the success of the learning outcomes established in each subject.

The educational community periodically conducts workshops, seminars, and other courses for the professional development of faculty members, regardless of their specialization ( Chukwuemeka & Samaila, 2020; Latorre-Cosculluela *et al*., 2023). Additionally, within the university environment, faculty members must remain constantly updated on technology-related topics and knowledge ( Alvites, 2019). In this context, a literature review has identified effective teaching practices with the aim of benefiting higher education institutions and selecting the best technology-related teaching strategies ( Khoza, 2022), leaving a gap in strategies and evaluations. However, when researching English teaching strategies in classrooms, six strategies were identified: memory, cognitive, compensatory, metacognitive, affective, and social. Researchers suggest further qualitative research in this area ( Tieocharoen & Rimkeeratikul, 2019).

Regarding contextual and pedagogical aspects, emerging pedagogical best practices in higher education address concerns related to assessment and improvement proposals at the end of the undergraduate thesis. However, there is a shortage of information on the methodological development of these best pedagogical practices ( Camilli Trujillo *et al*., 2022).

The updating and implementation of pedagogical approaches in university institutions create a connection between teaching and learning, positively impacting the quality of education and student engagement. A relevant methodological approach is the use of workshops as complements to the curriculum ( Asensio Pastor, 2019).

This research focuses on the systematization and analysis of effective pedagogical practices in the university context. Its importance lies in its direct impact on the quality of university education. These best practices not only enhance the student learning experience but also strengthen the ability of academic institutions to adapt to student needs. By understanding these practices, it is possible to promote and/or improve certain strategies, thus enhancing the quality of higher education. From this, the guiding question arises: “What innovative pedagogical practices have been developed in university settings to improve teaching and learning effectiveness?” This question involves the description of strategies and the evaluation of the pedagogical process. The research aims to systematize and provide scientific evidence for teaching professionals to use in their work and promote meaningful learning.

Regarding the design of activities and materials, current research indicates that the role of pedagogical practices and learning experiences presents challenges for both faculty and students in terms of necessary materials ( Tadesse *et al*., 2020). Furthermore, faculty members are always willing to introduce changes in teaching methodology ( Mataka *et al*., 2022). These changes or planned strategies are aimed at achieving academic goals, with the condition of achieving academic success through learning ( Ayu, 2021). Pedagogical practices emphasize the reflective aspect, not only for increasing knowledge but also for establishing a relationship between research and practice ( Sotomayor-Soloaga, 2021).

In Spain, research was conducted on the flipped classroom as a pedagogical strategy in higher education, concluding that it is necessary to combine the flipped classroom with gamification. This strategy improves academic performance and autonomy ( Carpena Arias & Esteve Mon, 2022). Similarly, research in Thailand explored the use of various strategies for language learning, revealing statistically significant differences in memory, cognitive, affective, and social strategies among students ( Tieocharoen & Rimkeeratikul, 2019).

A study conducted in Chile, focusing on teachers considered “the best teachers,” identified prominent characteristics such as promoting student autonomy, encouraging student participation, activating prior knowledge, and resolving doubts, strategically managing errors, creating a challenging thinking environment, asking good questions, making the most of time and space, having clarity in the evaluation system, and maintaining physical and emotional proximity ( Sotomayor-Soloaga, 2021).

Reviewing theories about best pedagogical practices reveals new subcategories based on teaching practices, allowing faculty to examine, interpret, and recreate their pedagogical practice, turning it into a source of learning from a perspective of change and innovation ( Merellano-Navarro *et al*., 2020).

The research highlights the need to use effective teaching strategies, suggesting that the debate method, among others, can improve students’ critical thinking ( Ahmod & Zhang, 2021; Crolla *et al*., 2019). Furthermore, the use of various strategies offers the opportunity to significantly transform the teaching process, promoting effective student learning ( Tadesse *et al*., 2021). Debate becomes a pedagogical tool for both faculty and students when providing quality feedback ( Crolla *et al*., 2019). Another way to actively participate in and collaborate in the learning process is through intrinsic or extrinsic motivation ( Tulyakul *et al*., 2022).

The literature review reveals dimensions of best pedagogical practices. Evaluation is present throughout the learning process and requires the presence of a methodology to achieve self-directed learning. It also requires faculty preparedness for various situations ( Porcher, 2020). Developing good pedagogical practices strengthens students’ critical thinking, enabling them to analyze information not only quantitatively but also qualitatively, complementing research ( Mahdi *et al*., 2020).

Likewise, technology and tools are necessary and are often used in classrooms through digital games ( Cavalcante-Pimentel *et al*., 2022). Finally, flexibility and adaptability are essential, as research results focus on the combination of the flipped classroom and gamification to improve motivation, academic performance, and autonomy of university students ( Carpena Arias & Esteve Mon, 2022). Another relevant aspect for improving pedagogical practice is international study trip experiences ( Ellinghaus *et al*., 2019).

Based on the theoretical foundations of Ausubel’s Meaningful Learning, which emphasizes that learning is most effective when it relates to meaningful prior knowledge, it is necessary to examine Mishra and Koehler’s TPACK model. This model describes the intersection of pedagogical, technological, and content knowledge necessary for effective teaching ( Vásconez Paredes, Darío & Mauricio, 2021).

Additionally, from this foundational structure, other forms of knowledge intersection emerge, including (a) Pedagogical Knowledge, which involves the fusion of pedagogical skills and experience; (b) Content Knowledge, which comprises a set of skills that teachers possess regarding the meaning of pedagogical content; (c) Technological Knowledge, which implies that faculty members stay updated and develop technological skills; (d) Pedagogical Content Knowledge, which involves not only having knowledge about a specific subject but also how to convey that knowledge; (e) Technological Pedagogical Knowledge, which integrates technology, pedagogical strategies, and pedagogical content; (f) Technological Content Knowledge, which involves integrating technology into the teaching of specific content; and (g) Technological Pedagogical Content Knowledge, which encompasses pedagogy, technology, and content ( Farhadi & Öztürk, 2023; Maipita *et al*., 2023).

Based on the presented theory, there is a need to delve deeper into the best pedagogical practices developed at the university level.

## Methods

The research follows a qualitative approach with a hermeneutic design. This design allows for an in-depth exploration and interpretation of various concepts presented by different authors, aiming to achieve a more comprehensive and enriched understanding. Furthermore, the research is classified as applied research since it works with existing elements but seeks to establish new concepts or methods to address the identified issue, aligning with the principles outlined in the Frascati Manual 2015 ( OECD, 2015).

Table 1 in the article presents the detailed procedures developed for each subcategory, providing a structured framework for the research methodology and analysis.

**Table 1.** Category and subcategories of good pedagogical practices.

Category	Subcategory
**Good pedagogical practice**	**Strategies**
It is a series of practices that teachers continuously carry out within the classroom, aiming to make the evaluation and learning process much more interactive for the internalization of new knowledge. Additionally, it possesses characteristics such as being innovative, effective, sustainable, and replicable ( Unesco, 2017).	Pedagogical strategies include frequent and personalized feedback. The strategy involves providing students with individualized feedback on their strengths and areas for improvement, as well as guidance for their academic and personal development.
	**Evaluation**
	Feedback is considered essential in the evaluation processes. In addition to providing grades or scores, it is recommended that teachers offer detailed feedback and guidance for improving performance. This helps students understand their mistakes, identify improvement strategies, and set realistic learning goals.

To identify relevant scientific articles, a specific set of keywords was carefully chosen. These keywords served as the initial search parameters, but the search was not limited to these terms alone. Synonyms and related terms were also incorporated to expand the scope of the search. Table 2 provides a comprehensive list of the selected keywords along with their corresponding synonyms. It is important to emphasize that these keywords represent the culmination of multiple search iterations, resulting in the formulation of a search ‘equation.’ This equation is designed to be replicable by fellow researchers in the future, thus contributing to the ongoing advancement of knowledge in the field.

**Table 2.** Synonyms of research keywords.

Research Keywords	Synonyms
Good pedagogical practices	Effective teaching strategies
university professors	Higher education teachers

Likewise, a search equation was employed, utilizing Boolean operators OR and AND. The OR operator was used for synonyms, while AND was employed to connect the keywords. The equation was structured as follows: (Good pedagogical practices OR Effective teaching strategies) AND (university professors OR Higher education teachers).

It is worth noting that the search was conducted across three databases. The first was the “ Educational Resources Information Center” (ERIC), specialized in Education. The second database was Scopus, known for its high-quality content for researchers, and finally, the Proquest database, accessed through advanced search functions, for potential future replication purposes.

To locate articles, a set of inclusion and exclusion criteria were applied. The initial criterion stipulated that articles must fall within the temporal range of 2015 to 2023. The decision to select this time frame was informed by the observation that there was no significant increase in scientific article production in the preceding decade (2005-2015). This suggests that research in this area began to gain relevance and increase in quantity starting from 2015 ( Gómez Velasco *et al*., 2022).

Furthermore, articles identified as conference papers and reviews were excluded due to their limited presence. Additionally, postgraduate work publications are infrequent and have limited impact on the international scientific community ( Mamani Benito *et al*., 2021).

It should be noted that duplicate articles across any of the three databases were removed. Finally, the authors conducted a thorough selection process, focusing on articles most pertinent to the research, based on the abstract and conclusion of each article. Table 3 provides a detailed overview of the procedures conducted.

**Table 3.** Inclusion and exclusion criteria for selected documents.

Database	Identification - Initial Search	Temporal Range - 10 years (2015-2023)	Eligibility - Subject - Education or Social Sciences/Peer-reviewed only/Full Text Availability	Inclusion - Higher Education/Publisher Restrictions/Teaching Methods/Articles	According to proposed objectives
Eric	204 040	71 988	9 915	1 615	15
Scopus	13	12	7	4	4
Proquest	222 334	54 366	19 980	19 457	6
Total	426 387	126 366	29 902	21 076	25

**Figure 1.**
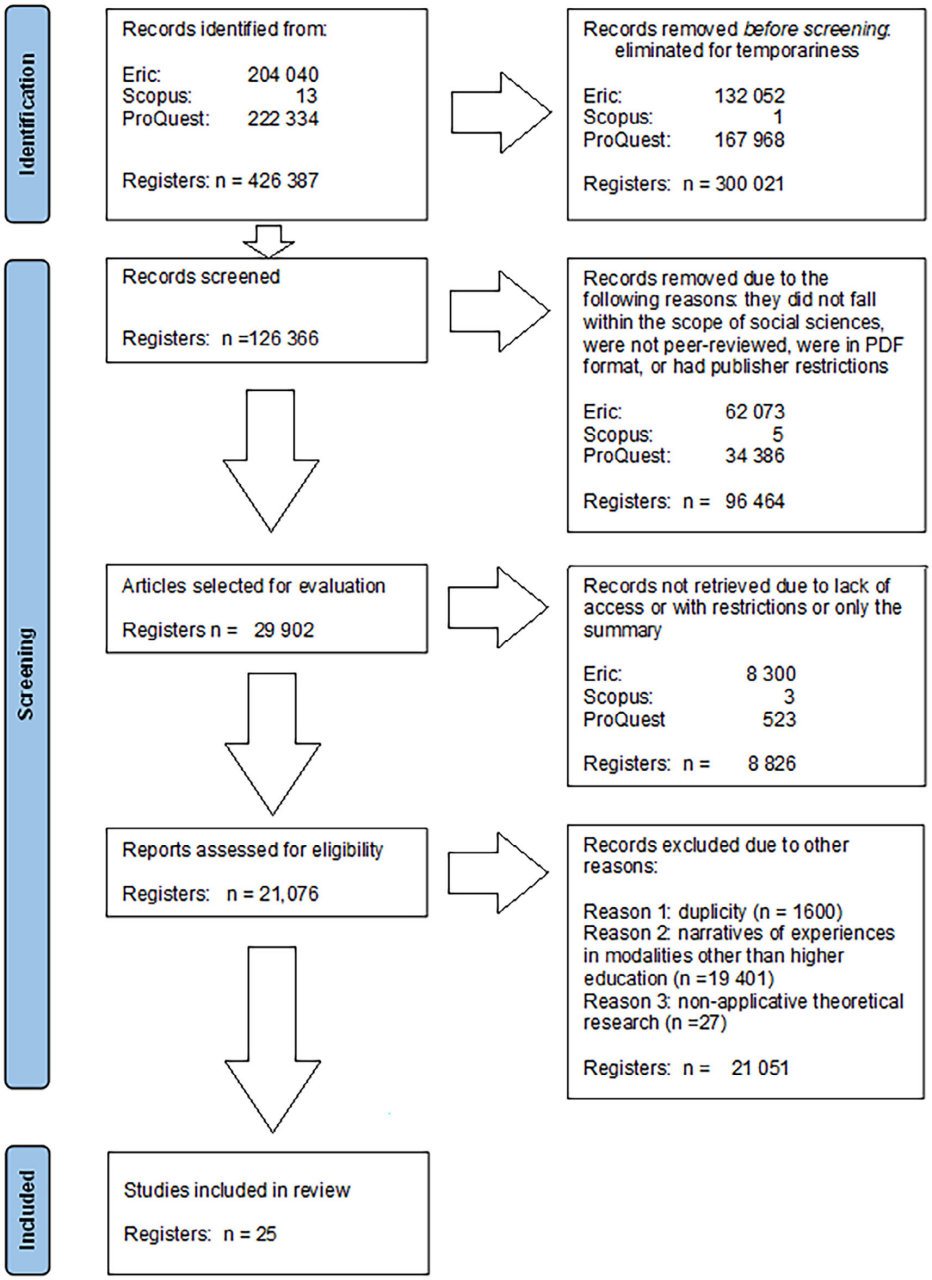
PRISMA diagram for the selection of scientific articles on good pedagogical practices.

Likewise, the PRISMA diagram was created, as shown in Figure 1, providing a graphical representation of the preselection process ( Page *et al*., 2021).

The research was structured in accordance with the PRISMA statement. In the methods section, the following criteria were taken into account: (a) Protocol and registration, (b) Eligibility criteria, (c) Sources of information, (d) Search strategy, (e) Study selection, (f) Data collection process, and (g) Data items ( Hutton *et al*., 2016). All of these points aim to adhere to the procedure outlined in the PRISMA statement, as illustrated in Table 4. A completed PRISMA checklist can be found as *Extended data* ( Suyo-Vega, Fernández-Bedoya, & Meneses-La-Riva, 2023).

**Table 4.** Methods for protocol, eligibility criteria, sources of information, search strategy, study selection, data collection process, and data items.

Method	
**Protocol**	The selected articles are archived in the Mendeley data management folder, following a coding system established for each database: SCOPUS-PRÁCTICASPP, SCIELO-PRÁCTICASPP, and ERIC-PRÁCTICASPP.
**Eligibility criteria**	Articles selected encompass the years 2015 to 2023; final-stage articles within the field of social sciences, peer-reviewed, freely accessible, and without publisher restrictions were chosen. Monographs, essays, editorials, and other materials not aligned with the research objectives were excluded.
**Information sources**	We selected scientific articles from the ERIC, Scopus, and Proquest databases. The search was conducted from May 1st to July 12th, 2023.
**Search equation**	We used the search equation (Good pedagogical practices OR Effective teaching strategies) AND (university professors OR Higher education teachers). In the Scopus database, this search yielded 13 results. After filtering by years, 12 articles were selected. Next, we filtered by the thematic area, keeping only those in the field of Social Sciences, resulting in 11 articles. We then removed 1 conference paper, leaving 10 eligible articles. Finally, we further refined the selection by choosing articles with open access, resulting in 4 articles. These 4 selected articles were downloaded and placed in a coded folder for analysis.
**Study selection**	The selected articles were initially analyzed by examining their abstracts, with a focus on identifying their objectives and activities related to good pedagogical practices. Articles that primarily constituted literature reviews or systematic reviews, or those that primarily defined or analyzed the importance of developing good practices, were excluded.
**Data collection process**	The collection process was carried out using Boolean operators, and the research team collaborated to identify the instances of good pedagogical practices within each article.
**Data items**	Each article was thoroughly reviewed in three aspects. The first aspect involved the analysis of informative data, such as the author’s name or names and the respective year of publication. The second aspect focused on identifying the instances of good pedagogical practices within the articles, including descriptions of the activities undertaken. The third aspect involved evaluating how the good pedagogical practice was presented or visualized. These three selected aspects were identified and documented in a table for better comprehension. The team analyzed the activities described in each research article and synthesized each finding through interpretation.

## Results

After the selection of articles, an in-depth analysis was conducted as detailed in Tables 5 and 6.

**Table 5.** Included studies.

Number	Reference	Journal	Good pedagogical practice	Design
1	( Ruiz de Gauna *et al*., 2015)	Educación Médica	Promotion of good practices based on competencies	Qualitative
2	( Zhong, 2018)	Journal of Higher Education Theory and Practice	Student-centered learning	Qualitative
3	( Zamora-Polo *et al*., 2019)	Education Sciences	Active learning pedagogy and gamification in a flipped classroom	Qualitative
4	( López Serrano, 2019)	El Futuro del Pasado	The use of cinema as a pedagogical approach	Mixed
5	( Medero & Albaladejo, 2020)	Knowledge Management and E-Learning	Creation of a wiki	Qualitative
6	( Jan *et al*., 2020)	Malaysian Journal of Learning and Instruction	Utilization of the LOTE (Languages Other Than English) pedagogy	Qualitative
7	( Isayeva *et al*., 2020)	Advanced Education	Strategies for teaching foreign languages through blended learning	Mixed
8	( du Plessis, 2020)	South African Journal of Education	Utilization of future teachers’ workbooks	Qualitative
9	( Foos, 2020)	Journal of Instructional Pedagogies	Micro-influencers for developing their personal brands	Qualitative
10	( Sun & Liu, 2021)	Higher Education Studies	Student-centered strategies	Qualitative
11	( Schwartzman *et al*., 2021)	RIED-Revista Iberoamericana de Educación a Distancia	Remote Assessment of Learning in the University	Qualitative
12	( Lindstrom *et al*., 2021)	Australian Journal of Teacher Education	Use of classroom scenarios for practicing difficult conversations	Qualitative
13	( Miranda *et al*., 2021)	Education Sciences	Peer observation	Mixed
14	( Galoyan *et al*., 2021)	Online Learning Journal	Online pedagogical practices that enhance transfer	Cuantitative
15	( Şeker & Inan Karagül, 2021)	Journal of Learning and Teaching in Digital Age	Self-directed learning strategies for effective writing through a self-assessment framework	Qualitative
16	( Sun & Liu, 2021)	Higher Education Studies	Effective strategies for implementing online teaching of theoretical mechanics	Qualitative
17	( Allison *et al*., 2021)	Journal of Higher Education Theory and Practice	Collaborative self-study	Qualitative
18	( Martín Gómez *et al*., 2022)	Revista Complutense de Educacion	The teaching journal, promoting a reflective and investigative attitude	Qualitative
19	( Fernández-Franco *et al*., 2022)	Estudios Pedagógicos	Development of reflective competence during physical education teacher training practices	Qualitative
20	( Fernández Miranda *et al*., 2022)	Bordón. Revista de Pedagogía	Inverted method as a didactic model in virtual learning	Cuantitative
21	( Donato *et al*., 2022)	Foro de Educación	Responsibility and pedagogical dialogue model, considering different learning styles as a positive resource	Qualitative
22	( Essa Aloud, 2022)	Arab World English Journal	Corrective online feedback	Qualitative
23	( Perdomo *et al*., 2022)	Learning and Teaching	Creative Process 3.0 and the use of digital tools	Mixed
24	( Camús Ferri *et al*., 2022)	Journal of Higher Education Theory and Practice	Dialogical literary gatherings	Qualitative
25	( Lobos *et al*., 2023)	Sustainability (Switzerland)	Lessons learned during remote teaching	Qualitative

**Table 6.** Analysis of developed strategies and the emerging impact resulting from the application of pedagogical best practices.

Number	Reference	Strategy	Type of strategy	Forms of assessment
1	( Ruiz de Gauna *et al*., 2015)	A paradigm shift involving changes not only in content, methodology, and assessment of teaching-learning processes, but also in those related to educational institutions, curriculum, and the culture of teachers/tutors.	1	Competency-based evaluation in specific situations.
2	( Zhong, 2018)	Improving communication between teachers and students using online tools within a large-scale class.	1	Ongoing student assessments and feedback favor the use of online courses.
3	( Zamora-Polo *et al*., 2019)	The strategy includes just-in-time teaching, an oral methodology, cooperative activities, and a game-based activity to generate positive emotions. It also involves the use of materials for home-based reading, such as videotutorials or podcasts.	2	Evaluation through post-session surveys in a Flipped classroom with gamification incentives.
4	( López Serrano, 2019)	A questionnaire was applied for a bibliographic survey containing titles of pedagogical movies, followed by the selection of the subject and student group to view them. In this context, cinema plays a central role.	2	Evaluation through cinema, with the school adapting to the digital and virtual society.
5	( Medero & Albaladejo, 2020)	A wiki was created to promote active collaboration and open education among students. The rules stipulated that only students could publish on this platform, although the public had free access. Continuous assessment and feedback were highlighted.	2,3	Evaluation based on the analysis of results obtained by students and teachers on the platform.
6	( Jan *et al*., 2020)	The LOTE (Languages Other Than English) pedagogy was developed through integrated teaching with technology and teacher narratives.	2	Evaluation under the understanding of language teaching practices (LOTE).
7	( Isayeva *et al*., 2020)	E-Learning was used to promote self-directed learning, with specific activities before or after in-class learning. This teaching approach was 40% electronic and 60% in-person, measuring literacy levels and linguistic competence.	2	Evaluation through questionnaires.
8	( du Plessis, 2020)	The strategy aimed to expose future education professionals to a “student-centered” approach by placing them in productive and successful educational institutions with different social backgrounds.	1	Evaluation based on three aspects: (a) participant perspectives, (b) author’s interpretations, and (c) theoretical framework.
9	( Foos, 2020)	The strategy involved students acting as micro-influencers to develop and promote their personal brands through social media and blogs.	2	Evaluation through messages on Slack and social networks.
10	( Sun & Liu, 2021)	Through the DingTalk platform, four strategies were developed to improve teaching: 1) live teaching, 2) the use of electronic whiteboards, 3) linking theory with practice, and 4) integrating curriculum content into ideological and moral education.	2	Formative evaluation using different methods such as class note review, asking questions, in-class exercises, and administering quizzes.
11	( Schwartzman *et al*., 2021)	A workshop with teachers identified learning focused on repetition and learning focused on comprehension. Forums were used as a means of communication.	1,2	Teacher evaluation to support or verify the learning processes.
12	( Lindstrom *et al*., 2021)	Instructors implemented classroom situations to help teacher trainees address difficult conversations with students, promoting critical reflection and discussion on creating culturally responsive classrooms, to change future teachers’ perceptions of effective teaching qualities.	1,3	Evaluation through debate and the proposal of new strategies.
13	( Miranda *et al*., 2021)	In the study, four higher education teachers from different disciplinary areas observed each other’s teaching practices. The goal was to assess whether constructive feedback focused on pedagogical practices rather than content. The importance of feedback was emphasized.	1,3	Evaluation through the comparison of quantitative and qualitative approaches.
14	( Galoyan *et al*., 2021)	The research focused on developing the transfer of topics related to online pedagogical practices across various contexts. It involved practice, feedback, fragmentation, and presentations.	2,3	Evaluation through metacognition.
15	( Şeker & Inan Karagül, 2021)	The methodology focused on completing a self-assessment framework with effective writing strategies, at three stages: before, during, and after writing. It described various strategies used in each case, such as brainstorming, grammar review tools like Grammarly, WhiteSmoke, LanguageTool, among others, and constant feedback.	2,3	Evaluation through semi-structured interviews.
16	( Sun & Liu, 2021)	The methodology consisted of identifying and describing reflective and investigative attitudes of future early childhood education teachers.	3	Reflective self-evaluation as a strategy.
17	( Allison *et al*., 2021)	Strategies used included active collaboration among students to build and share their knowledge.	1,3	Evaluation through reflection journals, course materials, class notes, and student responses and comments.
18	( Martín Gómez *et al*., 2022)	The methodology involved analyzing diaries of future teachers, from which emerged categories related to the educational context, teachers, students, and families, with a focus on teacher tasks.	1,3	Not specified.
19	( Fernández-Franco *et al*., 2022)	The methodology was divided into three phases: pre-active, where each student prepared their lesson in collaboration with a mentor and a cooperating teacher; simultaneously, researchers generated instruments such as observation rubrics and video cameras. The active phase involved all parties using a checklist and filming the process. Finally, the post-active phase involved filming the discussion and analyzing new knowledge about reflective practice.	2,3	Self-assessment, peer assessment, and through discussion groups.
20	( Fernández Miranda *et al*., 2022)	Implemented through a flipped classroom methodology using the Canva platform, communication occurred via email, internal messaging, chat, forums, and video conferencing, followed by forums and group tutorials.	2,3	Evaluation supported by feedback to achieve autonomous learning.
21	( Donato *et al*., 2022)	A new form of teaching outside the classroom was achieved through the creation of a radio program.	2,3	Evaluation and self-assessment in three phases: team self-assessment, peer evaluation at the end of the presentation, and teacher evaluation.
22	( Essa Aloud, 2022)	The strategy involved observing the classes of five teachers, conducting semi-structured interviews, and analyzing the knowledge and practices of the teachers. The research focused on corrective online feedback in an English oral expression class.	3	Evaluation through “output-prompting” strategies and less “input-providing.”
23	( Perdomo *et al*., 2022)	Pro.Seso Creativo® 3.0 is a methodology consisting of five phases designed to help students tackle challenges and problems to arrive at creative solutions.	2,3	Internal evaluations within discussion groups and end-of-semester surveys.
24	( Camús Ferri *et al*., 2022)	Interviews explored experiences with dialogical literary gatherings, highlighting the advantages and difficulties encountered, emphasizing cognitive and communication skills.	3	Not specified.
25	( Lobos *et al*., 2023)	A questionnaire was administered to gather the best learning experiences, including effective learning resources such as recorded classes, infographics and videos, and the use of relevant movies or TV series.	2,3	Three aspects were evaluated: (a) Types of feedback (individual, group, public, and anonymous); (b) moments of feedback (at the beginning of the class or immediately after execution), and (c) didactic feedback (capability or procedure).

Note: In Table 6, number 1 corresponds to student-centered teaching strategies. Number 2 corresponds to online teaching strategies and the use of educational technology. And number 3 corresponds to assessment and feedback strategies.

## Discussion

From the analyzed best practices, three key strategies that promote teaching and learning in higher education can be grouped as follows:

a) Student-Centered Teaching Strategies: In this approach, educators recognize the importance of actively involving students in their own learning process. Autonomy is valued, and individual student needs and learning styles are respected, fostering critical thinking ( Ahmod & Zhang, 2021; Crolla *et al*., 2019; Mahdi *et al*., 2020; Tadesse *et al*., 2021; Tulyakul *et al*., 2022).
b) Online Teaching Strategies and Educational Technology Usage: Faculty integrate educational technology and adapt it to online teaching environments, utilizing digital tools to enhance teaching and learning. This turns learning into a source of pedagogical innovation ( Carpena Arias & Esteve Mon, 2022; Cavalcante-Pimentel *et al*., 2022; Merellano-Navarro *et al*., 2020).
c) Assessment and Feedback Strategies: These strategies enable educators to provide constructive and ongoing feedback to students with the aim of improving learning and academic growth ( Maipita *et al*., 2023; Vásconez Paredes, Darío & Mauricio, 2021).

In conclusion, promoting pedagogical best practices based on student-centered strategies and self-directed learning is essential for improving the teaching and learning process in higher education, beyond traditional teaching methods. These practices allow educators to tailor instruction to student needs and learning styles, promoting student autonomy and motivation.

Incorporating strategies using technological tools makes teaching effective and enriches the learning experience in virtual environments within the university setting. Additionally, the use of digital tools and innovative methodologies facilitates access to education and provides rapid and effective feedback to students.

Research on pedagogical best practices in higher education is essential for the advancement of teaching and learning. It provides valuable information that benefits both current and future educators, equipping them with tools to address challenges and adapt strategies to their context. Moreover, it contributes to the continuous improvement of teacher training and fosters innovation in the educational sphere, creating a more stimulating and effective environment for the teaching and learning process.

Certain limitations were identified during the research process. One limitation is related to the timeframe. The research was conducted between 2015 and 2023. However, to gain a more comprehensive and longitudinal view of trends in the literature, it would be beneficial to extend the study period by several additional years.

Another limitation is related to the availability of sources. Despite having access to a variety of databases such as ERIC, SciELO, and Scopus, it has been observed that some research of interest is not available in full PDF format, hindering the sharing of certain findings with the scientific community.

This research significantly contributes to existing knowledge in the field of pedagogical best practices in the university context. Through document analysis, online teaching and student-centered learning, two fundamental aspects of modern pedagogy, were addressed.

The research team proposes alternatives such as the implementation of a continuous professional development program for faculty, designed to be accessible virtually and on an ongoing basis. This initiative aims to ensure that faculty members are in a constant state of development and skill updating, ultimately translating into a high-quality learning experience for students.

In this context, it is recommended to expand research to other modalities and educational levels, as well as to explore successful experiences in countries that have effectively adopted these pedagogical practices.

## Data Availability

All data underlying the results are available as part of the article and no additional source data are required. Zenodo: PRISMA 2020 checklist for ‘Beyond Traditional Teaching: A Systematic Review of Innovative Pedagogical Practices in Higher Education’ https://doi.org/10.5281/zenodo.8404402 ( Suyo-Vega, Fernández-Bedoya, & Meneses-La-Riva, 2023). Data are available under the terms of the Creative Commons Attribution 4.0 International license (CC-BY 4.0).
