# Supplementary material for: Beyond traditional teaching: a systematic review of innovative pedagogical practices in higher education
Source: F1000Res. 2024 Dec 17;13:22. Originally published 2024 Jan 8. [Version 2] doi: 10.12688/f1000research.143392.2 (PMC11795252; doi:10.12688/f1000research.143392.2)
Supplement: Peer review history

# Reviewer report: Reviewer response for version 2

**DOI:** 10.5256/f1000research.175777.r349763

**Published:** 2025-01-20

**Authors:** Maria Alfredo Moreira

**Affiliations:** 1 Research Centre on Education, University of Minho, Minho, Portugal

As stated in my earlier review to this text, the mais problem lies in its theoretical underpinning. This issue was not addressed in the authors' response and it lingers in the revised version as it states (my underlining): "The research is grounded in Ausubel’s Meaningful Learning theory, which emphasizes the importance of connecting new knowledge with prior meaningful concepts to achieve deep learning. Similarly, Mishra and Koehler’s TPACK model is key to analyzing the intersection of pedagogical, technological, and content knowledge in best teaching practices. These theories frame the analysis, enabling the integration of innovative teaching strategies in specific contexts and contributing to the advancement of the state of the art in higher education." I expected this issue to be addressed but it was not.

 Another issue was related to the focus on Social Sciences. Now it appears grounded in Durkheim's concept of education, but social sciences are not restricted to education. This choice is still not clarified.

 In addition, limitations are addressed in the Discussion section, even though there is a specific section for it. In this section (Limitations), the paragraph on the data bases justifies the choice made, so it seems to be part of the methodology section. The same goes to the choice of the languages and for the design of the study. These are methodological choices that can be made, not limitations per se. The discussion section synthesises results in groups a) and b), but not c). The results are not given detailed attention, but they are the main asset of the text - what do we know now that we did not know before? The three goups of results are well-known for some decades now - is there anything new?, including the importance of a savvy pedagogical use of technologies.

Are the rationale for, and objectives of, the Systematic Review clearly stated?

Partly

Is the statistical analysis and its interpretation appropriate?

Not applicable

If this is a Living Systematic Review, is the ‘living’ method appropriate and is the search schedule clearly defined and justified? (‘Living Systematic Review’ or a variation of this term should be included in the title.)

Not applicable

Are sufficient details of the methods and analysis provided to allow replication by others?

Yes

Are the conclusions drawn adequately supported by the results presented in the review?

Partly

Reviewer Expertise:

Language Education; Teacher Education; Pedagogy in Higher Education

I confirm that I have read this submission and believe that I have an appropriate level of expertise to state that I do not consider it to be of an acceptable scientific standard, for reasons outlined above.

# Reviewer report: Reviewer response for version 2

**DOI:** 10.5256/f1000research.175777.r349762

**Published:** 2025-01-09

**Authors:** Budi Waluyo

**Affiliations:** 1 Walailak University, Thai Buri, Nakhon Si Thammarat, Thailand

Dear Author(s),

 Thank you for revising the manuscript. I have carefully read your revised version. However, the quality has not improved significantly. Below are my comments:

1. **Background of the Abstract**: You can condense this to one sentence. However, you need to revise the first sentence to emphasize the research gap, which will help readers understand why this research was necessary.
2. **Method of the Abstract**: There is no need to include the research question in this section.
3. **Results of the Abstract**: You need to mention the analysis method you used and briefly explain how you performed it.
4. **Introduction Section**: In the first paragraph, you should briefly discuss the development of research on innovative pedagogical practices in higher education. Then, briefly elaborate on what previous studies have explored and what they have not, which will lead to the contribution your study intends to make. In its current form, the first paragraph of your introduction is neither appealing nor convincing. First, it lacks references, which is crucial in this important section. Second, it is too short.
5. **Overall Introduction**: After reading the entire introduction, I find it incoherent and unconvincing.
  a) It is unclear what exactly you are trying to introduce in this section.
  b) You mention "literature review" several times, but it is unclear whether you are referring to your own literature review or other studies.
  c) The introduction seems disorganized and difficult to understand. You need to reorganize and rewrite this section. You should focus on your topic, which is innovative pedagogical practices in higher education.
  - Why is this topic worth exploring through a literature review?
  - What has been explored in previous studies?
  - What distinctive contributions does your study aim to make?
    In its current form, the section is too unfocused, and it may be difficult for readers to understand. An extensive revision is needed.
6. **Method Section**: The method section looks fine overall, but I have one suggestion: please revise the paragraphs. Each paragraph should consist of at least 7-8 sentences, not just 2. You have many very short paragraphs throughout the article. Please adjust this accordingly.
7. **Results Section**: This section needs significant improvement. It mainly consists of tables, with little explanation.
  a) Since you have a research question, you should elaborate on the results in relation to it. Even though you include tables, you need to discuss the results in a critical and comprehensive manner. A thorough revision is needed for this section.
8. **Discussion Section**: This section is too brief and lacks depth. Your discussion basically only consists of points a, b, and c. They are not critical and comprehensive enough.
  - You need to discuss how your findings relate to previous research.
  - You should elaborate on the implications of your findings, and so on.
  - Additionally, you need to create a specific sub-section for the conclusion.

Overall, you need to do major revisions to the introduction, result, and discussion sections, among others. 

 Best,

 Reviewer

Are the rationale for, and objectives of, the Systematic Review clearly stated?

Partly

Is the statistical analysis and its interpretation appropriate?

Partly

If this is a Living Systematic Review, is the ‘living’ method appropriate and is the search schedule clearly defined and justified? (‘Living Systematic Review’ or a variation of this term should be included in the title.)

No

Are sufficient details of the methods and analysis provided to allow replication by others?

Partly

Are the conclusions drawn adequately supported by the results presented in the review?

Partly

Reviewer Expertise:

ELT, Education, and Education Technology.

I confirm that I have read this submission and believe that I have an appropriate level of expertise to state that I do not consider it to be of an acceptable scientific standard, for reasons outlined above.

# Reviewer report: Reviewer response for version 2

**DOI:** 10.5256/f1000research.175777.r349760

**Published:** 2025-01-07

**Authors:** Jay Fie Paler Luzano

**Affiliations:** 1 Bukidnon State University, Malaybalay City, Bukidnon, Philippines

The study already complied with the required scientific rigor.

Are the rationale for, and objectives of, the Systematic Review clearly stated?

Partly

Is the statistical analysis and its interpretation appropriate?

Partly

If this is a Living Systematic Review, is the ‘living’ method appropriate and is the search schedule clearly defined and justified? (‘Living Systematic Review’ or a variation of this term should be included in the title.)

Partly

Are sufficient details of the methods and analysis provided to allow replication by others?

Partly

Are the conclusions drawn adequately supported by the results presented in the review?

Partly

Reviewer Expertise:

Mathematics Education

I confirm that I have read this submission and believe that I have an appropriate level of expertise to confirm that it is of an acceptable scientific standard.

# Reviewer report: Reviewer response for version 1

**DOI:** 10.5256/f1000research.157048.r301479

**Published:** 2024-08-07

**Authors:** Maria Alfredo Moreira

**Affiliations:** 1 Research Centre on Education, University of Minho, Minho, Portugal

The text reports a systematic review study focusing on a critical aspect of higher education quality, as is pedagogical practices. It addresses a relevant research question ("What innovative pedagogical practices have been developed in university settings to improve teaching and learning effectiveness?”), a question that "involves the description of strategies and the evaluation of the pedagogical process.". It partially achieves its aim, of "systematiz[ing] and provide scientific evidence for teaching professionals to use in their work and promote meaningful learning." The methodology is sound, well-laid out and with a clear description of the path that was followed in order to achieve its results.

 However, the Introduction does not address in a systematic and coherent manner the key concepts of the study: Good pedagogical practices AND effective teaching strategies. The studies that sustain this section seem to have been selected rather randomly, as it goes into language teaching details (listing language learning strategies as if they would be a synomym of pedagogical strategies, for example), several countries (like Spain, Chile or Thailand - why these countries?), or learning theories (Ausubel's, MIshra & Koehler - what is their importance for defining the key constructs? How were these key constructs used in interpreting the results?). It is not clear how the state of the art outlined in this section will be expanded or enriched by the study.  As the Discussion section does not confront the results with the concepts in the introduction, it is not clear what the advancement in the field was. 

 Even though the methodology is sound, there are minor issues that would improve the text. Only by reading Figure 1 the reader becomes aware that the focus is on social sciences (why this focus?), as it is neither stated in the research question nor in the introduction. And why were systematic reviews excluded? Figure 1 reports exclusion of pdf formats but in the discussion it is stated: "it has been observed that some research of interest is not available in full PDF format". Thus, it is not clear whether the format of the text was a criterion for exclusion or not.

Are the rationale for, and objectives of, the Systematic Review clearly stated?

Partly

Is the statistical analysis and its interpretation appropriate?

Not applicable

If this is a Living Systematic Review, is the ‘living’ method appropriate and is the search schedule clearly defined and justified? (‘Living Systematic Review’ or a variation of this term should be included in the title.)

Not applicable

Are sufficient details of the methods and analysis provided to allow replication by others?

Yes

Are the conclusions drawn adequately supported by the results presented in the review?

Partly

Reviewer Expertise:

Language Education; Teacher Education; Pedagogy in Higher Education

I confirm that I have read this submission and believe that I have an appropriate level of expertise to confirm that it is of an acceptable scientific standard, however I have significant reservations, as outlined above.

# Response

**Published:** 2024-12-09

**Authors:** VICTOR HUGO FERNANDEZ BEDOYA

**Affiliations:** Universidad Cesar Vallejo, Trujillo, La Libertad, Peru

Dear reviewer:

 Thank you for your comments, we have managed to make improvements in the new version of the manuscript, which will be online in the following days. Meanwhile, we attach the responses to each of the observations.

 Regarding the Specific Objectives

 The general objective of the study was to systematize innovative pedagogical best practices in the university context.

 Additionally, the specific objectives included:

1. **Analyzing pedagogical best practices** alongside their respective methodological approaches in the university setting.
2. **Examining the applied strategies**, focusing on the types of strategies (student-centered, online teaching or technology-based, and evaluation or feedback strategies).
3. **Evaluating the methods used to assess pedagogical practices**, ensuring a comprehensive understanding of their effectiveness.

# Reviewer report: Reviewer response for version 1

**DOI:** 10.5256/f1000research.157048.r301470

**Published:** 2024-07-24

**Authors:** Hoang-Yen Phuong

**Affiliations:** 1 School of Foreign Languages, Can Tho University, Can Tho, Can Tho, Vietnam

The review has several limitations that may affect its comprehensiveness and depth. Covering articles only from 2015 to 2023, it might miss earlier developments or long-term trends in pedagogical practices. Although it includes publications in Spanish and English, it could still overlook important research in other languages, leading to potential language bias. The reliance on three databases, despite their reputability, might not capture all relevant studies, and the lack of a formal quality assessment of the included studies is a notable omission in a systematic review.

 Furthermore, the review primarily offers qualitative insights without conducting a meta-analysis or robust statistical analysis, which could have strengthened the evidence for the identified practices. It also has a broad focus on all innovative practices in higher education, potentially sacrificing depth in specific areas. The limited discussion of contradictory findings and the absence of detailed exploration of how practices vary across different countries or cultural contexts restrict the review's ability to provide a balanced and comprehensive view.

 Other weaknesses include the omission of student perspectives and outcomes, which are crucial for understanding the effectiveness of teaching practices. The authors acknowledge limited access to full-text articles, which might have excluded relevant studies, and they do not address potential publication bias, possibly skewing the results toward positive findings. Finally, the review does not extensively discuss the challenges and barriers to implementing innovative practices in various higher education settings, limiting its practical applicability.

Are the rationale for, and objectives of, the Systematic Review clearly stated?

Yes

Is the statistical analysis and its interpretation appropriate?

Partly

If this is a Living Systematic Review, is the ‘living’ method appropriate and is the search schedule clearly defined and justified? (‘Living Systematic Review’ or a variation of this term should be included in the title.)

Partly

Are sufficient details of the methods and analysis provided to allow replication by others?

Partly

Are the conclusions drawn adequately supported by the results presented in the review?

Partly

Reviewer Expertise:

TESOL, testing and assessment, higher education

I confirm that I have read this submission and believe that I have an appropriate level of expertise to confirm that it is of an acceptable scientific standard, however I have significant reservations, as outlined above.

# Response

**Published:** 2024-12-09

**Authors:** VICTOR HUGO FERNANDEZ BEDOYA

**Affiliations:** Universidad Cesar Vallejo, Trujillo, La Libertad, Peru

Dear Reviewer:

 Thank you for your comments, we have managed to make improvements in the new version of the manuscript, which will be online in the following days. Meanwhile, we attach the responses to each of the observations.

**Regarding the Timeframe (2015 to 2023)**

 According to the UNESCO report (2015), the global education landscape experienced a radical transformation in methods, content, and learning spaces. This transformation has significantly impacted higher education. Consequently, the need for a shift in pedagogical practices emerged, gaining momentum in 2015 in response to demands for more flexible, student-centered education adapted to the digital age (UNESCO, 2015). Additionally, the decision to focus on this period was based on the observation that the preceding decade (2005–2015) did not see a significant increase in the scientific production of articles. This suggests that research began to gain relevance and increase in volume starting in 2015 (Gómez Velasco et al., 2022).

**Regarding the Language**

 Articles in English and Spanish were analyzed due to the predominance of these languages in the selected databases, such as SciELO, Scopus, and ERIC. These platforms are recognized for their focus on disseminating research in these languages, facilitating access to a wide range of scientific literature (Márquez Jiménez & Alcántara Santuario, 2017).

 Regarding the Three Databases

- **SciELO**: This database was selected because it democratizes access to scientific knowledge in Latin America, highlighting regional research previously considered "lost science" due to limited distribution (Crespo Fajardo, 2019).
- **Scopus**: Chosen for its broad international coverage and reputation as a key resource for evaluating scientific output. Scopus includes a large proportion of articles in English (67%) and Spanish (86%), providing access to impactful and relevant research across various disciplines (Márquez Jiménez & Alcántara Santuario, 2017).
- **ERIC**: Selected for its specialization in education worldwide and its provision of access to relevant literature in the field (Mosquera Ayala, 2017).

**Regarding Meta-Analyses or Solid Statistical Methods**

 The research aimed to systematize innovative pedagogical practices in higher education, which aligns more closely with a systematic review than a meta-analysis. Meta-analyses focus on quantifying effects using numerical data. Additionally, many of the studies reviewed did not provide sufficient or consistent data, such as means and standard deviations, necessary for conducting a meta-analysis (Debby Ng et al., 2022).

**Regarding the Lack of Comparisons Between Countries**

 The main objective of the research was to systematize best pedagogical practices in higher education, identifying effective strategies in the academic field. This required a detailed analysis of key elements, such as pedagogical approaches, types of strategies, and evaluation methods, without cross-country comparisons being part of the initial design. This methodological decision was based on the goal of identifying general patterns applicable on a global scale rather than focusing on local specificities. Additionally, systematic reviews must clearly define their scope to ensure analytical coherence and avoid excessive dispersion of results (Grant & Booth, 2009).

**Regarding Challenges and Barriers to Implementing Innovative Practices**

 The decision not to address challenges in detail was due to the limited scope of this research.

# Reviewer report: Reviewer response for version 1

**DOI:** 10.5256/f1000research.157048.r291882

**Published:** 2024-07-02

**Authors:** Jay Fie Paler Luzano

**Affiliations:** 1 Bukidnon State University, Malaybalay City, Bukidnon, Philippines

The rationale for and objectives of the systematic review are partly stated. The study aims to synthesize innovative pedagogical best practices within the university context to improve teaching and learning effectiveness. However, there may be room for further clarification or expansion on the specific objectives.

 Sufficient details of the methods and analysis are partly provided to allow replication by others. The study followed the PRISMA methodology, including criteria such as protocol, eligibility criteria, sources of information, search strategy, study selection, data collection process, and data items . This structured approach enhances the replicability of the study.

 The review did not specifically mention statistical analysis, as it focused more on synthesizing innovative pedagogical practices rather than statistical data analysis . Therefore, the appropriateness of statistical analysis and its interpretation is not applicable in this context.

 The conclusions drawn in the review are supported by the results presented, which include an in-depth analysis of selected articles and the impact of innovative pedagogical practices in higher education. The study aimed to delve deeply into the strategies utilized, pedagogical approaches adopted, and the noticeable impact achieved after their implementation, which supports the conclusions drawn.

Are the rationale for, and objectives of, the Systematic Review clearly stated?

Partly

Is the statistical analysis and its interpretation appropriate?

Partly

If this is a Living Systematic Review, is the ‘living’ method appropriate and is the search schedule clearly defined and justified? (‘Living Systematic Review’ or a variation of this term should be included in the title.)

Partly

Are sufficient details of the methods and analysis provided to allow replication by others?

Partly

Are the conclusions drawn adequately supported by the results presented in the review?

Partly

Reviewer Expertise:

Mathematics Education

I confirm that I have read this submission and believe that I have an appropriate level of expertise to confirm that it is of an acceptable scientific standard, however I have significant reservations, as outlined above.

# Response

**Published:** 2024-12-09

**Authors:** VICTOR HUGO FERNANDEZ BEDOYA

**Affiliations:** Universidad Cesar Vallejo, Trujillo, La Libertad, Peru

Thank you for your comments, we have managed to make improvements in the new version of the manuscript, which will be online in the following days. Meanwhile, we attach the responses to each of the observations.

**Regarding the Selected Countries**

 The choice of countries was not random; the selection was intentional because they represent diverse educational contexts across Europe, Asia, and America. The choice of Spain, Thailand, and Chile was based on their relevance in recent research exploring various aspects of effective pedagogical practices across different cultural and educational settings. The studies from these countries allow for an analysis of effective strategies that have been adopted and evolved according to their unique characteristics.

 For example, research conducted in Thailand (Tieocharoen & Rimkeeratikul, 2019) focuses on analyzing language learning strategies, such as memory, cognitive, affective, and social strategies. Although these strategies pertain to the student’s domain, the study illustrates effective pedagogical strategies.

**Regarding Learning Theories and Their Importance**

 This research is grounded in **Ausubel’s Meaningful Learning Theory**, which emphasizes the importance of connecting new knowledge with meaningful prior concepts to achieve deep learning. Furthermore, **Mishra and Koehler’s TPACK Model** is essential for analyzing the intersection of pedagogical, technological, and content knowledge within effective teaching practices. These theories frame the analysis by allowing the integration of innovative teaching strategies within specific contexts, thereby contributing to advancements in higher education research.

**Regarding the Expanded State of the Art**

 The research does not merely summarize the state of the art but also expands it by analyzing key theories within the university context. This approach provides added value by offering an updated and nuanced understanding of the topic to address contemporary pedagogical challenges. Furthermore, by identifying the application of these theories in classroom settings, this study contributes evidence to enrich existing knowledge about pedagogy and content in higher education teaching.

**Regarding Theories and Discussion**

 Strategies such as **debate and gamification** reflect Ausubel’s principle of connecting new learning to prior significant knowledge, facilitating the assimilation and application of knowledge. Similarly, the inclusion of technology and pedagogical tools aligns with the **TPACK Model**, which emphasizes the integration of pedagogical, technological, and content knowledge to enhance teaching practices.

**Regarding Why This Research is Categorized Within Social Sciences**

 According to Émile Durkheim, **education is a key social phenomenon in the reproduction and transformation of society.** It plays a fundamental role in the socialization of individuals and in the transmission of norms, values, and knowledge that ensure social cohesion. Therefore, research on **pedagogical practices** falls within the realm of social sciences because it influences the formation of individuals within a social context (Durkheim, 2015).

**Regarding the Exclusion of Systematic Reviews**

 The systematic reviews were excluded from the results section because this research focused on empirical studies—both quantitative quasi-experimental designs and qualitative case studies—that directly addressed innovative pedagogical practices. Although systematic reviews are valuable, they do not align with the study's aim of obtaining primary and context-specific data on the practical application of these strategies. Therefore, the research prioritized original studies that provided direct evidence and practical insights into pedagogical practices.

 Additionally, during the article review phase, documents in PDF format were identified, but in some cases, only titles and abstracts were available rather than full texts. Despite access to well-recognized databases such as ERIC, SciELO, and Scopus, restrictions in editorial access limited the inclusion of some relevant research. These constraints are highlighted under the **Limitations** section.

# Reviewer report: Reviewer response for version 1

**DOI:** 10.5256/f1000research.157048.r268905

**Published:** 2024-05-10

**Authors:** Budi Waluyo

**Affiliations:** 1 Walailak University, Thai Buri, Nakhon Si Thammarat, Thailand

Dear Authors, 

 First, I have seen frequently utilized vocabulary in generative AI applications. If you utilize any of these applications, it is essential to acknowledge and elucidate the extent to which they were employed in the composition.

 Second, the initial paragraph of the introduction holds paramount significance as it serves to inform the readers about the evolution of the research domain, the scope of prior investigations, and the unique addition of your study. Nevertheless, your initial paragraph lacks any of these vital elements. You should modify it in accordance with the necessary changes.

 Furthermore, your introduction lacks criticality and persuasiveness. To enhance the clarity of the current literature and highlight the contribution of your study, it is necessary to critically synthesize the findings of relevant studies.

 Third, it is imperative to formulate research questions in order to provide a clear focus for your research and facilitate the comprehension of your reported findings by readers.

 Fourth, it is necessary to include a literature review section in order to establish robust foundational frameworks. This style of study appears to be lacking in strength. 

 Fifth, it is necessary for you to provide more detailed information about the results in paragraphs, not just showing tables.

 Sixth, your analysis lacks sufficient depth and fails to adequately engage with the current related literature.

 According to these six factors, it is necessary for you to make significant revisions to this manuscript.

Are the rationale for, and objectives of, the Systematic Review clearly stated?

Partly

Is the statistical analysis and its interpretation appropriate?

Partly

If this is a Living Systematic Review, is the ‘living’ method appropriate and is the search schedule clearly defined and justified? (‘Living Systematic Review’ or a variation of this term should be included in the title.)

No

Are sufficient details of the methods and analysis provided to allow replication by others?

Partly

Are the conclusions drawn adequately supported by the results presented in the review?

Partly

Reviewer Expertise:

ELT, Education, and Education Technology.

I confirm that I have read this submission and believe that I have an appropriate level of expertise to state that I do not consider it to be of an acceptable scientific standard, for reasons outlined above.
